# Higher serum uric acid levels are associated with improved outcomes in acute ischemic stroke patients following intravenous thrombolysis with alteplase—a retrospective cohort study

**DOI:** 10.3389/fneur.2026.1759799

**Published:** 2026-03-17

**Authors:** Jingxian Ni, Jiahong Huang, Xiaoming Rong, Weike Zeng, Jialu Lin, Ying Peng, Xiangpen Li, Jingrui Pan

**Affiliations:** 1 Department of Neurology, Sun Yat-sen Memorial Hospital, Sun Yat-sen University, Guangzhou, China; 2 Shenshan Medical Center, Sun Yat-sen Memorial Hospital, Sun Yat-sen University, Shanwei, China; 3 Department of Radiology, Sun Yat-sen Memorial Hospital, Sun Yat-sen University, Guangzhou, China; 4 Guangdong Provincial Key Laboratory of Malignant Tumor Epigenetics and Gene Regulation, Sun Yat-sen Memorial Hospital, Sun Yat-sen University, Guangzhou, China

**Keywords:** acute ischemic stroke, alteplase, intravenous thrombolysis, outcome, serum uric acid

## Abstract

**Background:**

Emerging evidence suggests that hyperuricemia may serve as a predictor of favorable outcomes in acute ischemic stroke (AIS) patients receiving endovascular treatment; however, the relationship between serum uric acid (SUA) levels and clinical outcomes in AIS patients treated with intravenous thrombolysis (IVT) remains underexplored.

**Methods:**

In this retrospective study, we analyzed AIS patients who underwent IVT with alteplase within 4.5 h of symptom onset, excluding those undergoing subsequent endovascular intervention, at our hospital between November 2021 and December 2024. Data collection encompassed baseline demographic and clinical characteristics, SUA levels measured within 24 h post-thrombolysis, and neuroimaging findings from cranial computed tomography and magnetic resonance scans. The primary outcome was defined as an excellent 90-day functional outcome, characterized by a modified Rankin Scale (mRS) score of 0–1.

**Results:**

Among 194 screened patients, 130 were included in the final analysis. Of these, 87 patients (66.92%) achieved an excellent 90-day outcome. Multivariable logistic regression analysis, adjusted for potential confounders, revealed that patients with higher SUA levels (>360 μmol/L) exhibited a significantly higher likelihood of achieving an excellent 90-day outcome (adjusted OR: 2.690, 95% CI: 1.082–6.685, *p* = 0.033) and a favorable 90-day outcome (mRS 0–2) (adjusted OR: 4.271, 95% CI: 1.438–12.686, *p* = 0.009) compared to those with normal SUA levels. No significant association was observed between SUA levels and the incidence of intracranial hemorrhage or 90-day mortality (*p* > 0.05).

**Conclusion:**

These findings indicate that higher SUA levels are significantly associated with improved 90-day functional outcomes in AIS patients treated with alteplase thrombolysis within 4.5 h of symptom onset.

## 1 Introduction

Ischemic stroke, constituting over 70% of all stroke cases, represents a predominant contributor to mortality and disability in China (1, 2). Intravenous thrombolysis (IVT) utilizing alteplase has been established as the standard therapeutic intervention for eligible patients presenting with acute ischemic stroke (AIS), as endorsed by both Chinese (3) and international guidelines (4). Nevertheless, approximately 40% of patients fail to achieve functional independence at 90 days post-IVT, even when administered within the 4.5-h time window after symptom onset (5). Several risk factors associated with unfavorable outcomes have been identified, including advanced age, elevated baseline National Institutes of Health Stroke Scale (NIHSS) scores, prolonged onset-to-thrombolysis (OTT) intervals, diabetes mellitus, and pre-thrombolysis prothrombin time (6). However, the identification of potential patients who are most likely to derive optimal therapeutic benefits from IVT still remains challenging, suggesting that the predictive utility of potential biomarkers for clinical outcomes remains inconclusive.

Hyperuricemia has emerged as a prevalent comorbidity of ischemic stroke in China, with its prevalence among Chinese adults increasing from 11% to 14% between 2015 and 2019 (7). This rise is particularly pronounced in coastal regions and among male populations, likely driven by adverse dietary changes (e.g., increased consumption of alcohol, purine-rich foods, and sugar-sweetened beverages) and the growing burden of obesity and metabolic syndrome (8). Clinical researches have demonstrated a significant association between hyperuricemia and an elevated incidence of stroke, particularly ischemic stroke (9–11). Additionally, a prospective study revealed that increased serum uric acid (SUA) levels were associated with improved outcomes following endovascular treatment in AIS patients (12). Moreover, uric acid supplementation has been suggested to mitigate early clinical deterioration in AIS patients undergoing IVT treatment with alteplase within 4.5 h of symptom onset (13). Nevertheless, the relationship between SUA levels and clinical outcomes in AIS patients following alteplase thrombolysis without subsequent endovascular intervention remains undetermined. Consequently, this study aims to investigate the impact of higher SUA levels on 90-day functional outcomes in AIS patients eligible for IVT within 4.5 h of onset, while excluding those who underwent endovascular treatment, as this cohort has been previously examined in another study (12).

## 2 Methods

### 2.1 Study design and patients

This single-center retrospective observational study was approved by the Institutional Review Board of Shenshan Medical Center, Sun Yat-sen Memorial Hospital, Sun Yat-sen University (Protocol No. 2024-SSKY-219-01). The study cohort consisted of consecutive AIS patients who received intravenous thrombolysis with alteplase between November 2021 and December 2024. Inclusion criteria were defined as: (1) age >18 years; (2) confirmed AIS diagnosis within 4.5 h of symptom onset; (3) treatment with alteplase-based IVT; (4) availability of SUA measurements within 24 h post-IVT; and (5) documented modified Rankin Scale (mRS) scores (14) at 90-day follow-up. Exclusion criteria included: (1) premorbid mRS score ≥3; (2) administration of alternative thrombolytic agents (e.g., tenecteplase); (3) subsequent endovascular intervention following IVT; (4) concurrent diagnosis of malignant neoplasms; or (5) loss to follow-up at 90 days.

### 2.2 Clinical treatment

Intravenous alteplase was administered at a standardized dose of 0.9 mg/kg (capped at a maximum of 90 mg) within a therapeutic window of 4.5 h following the onset of stroke symptoms when clinically indicated, with an initial bolus injection comprising 10% of the total calculated dose, followed by a continuous infusion of the remaining 90% over a 60-min period. Cranial computed tomography (CT) was performed 24 h post-IVT, followed by cranial magnetic resonance imaging (MRI) within 7 days to assess the presence of intracranial hemorrhage (ICH). The prescription of antiplatelet or anticoagulant agents, statins, antihypertensive medications, and hypoglycemic drugs adhered to the established guidelines for stroke prevention (15).

### 2.3 Data collection

The baseline was operationally defined as data collected within the initial 24-h period following hospital admission. Comprehensive baseline data were systematically extracted from electronic medical records, encompassing demographic characteristics, cardiovascular disease history, vascular risk factors, and prior medication use, with particular attention to agents potentially influencing SUA levels. Laboratory analyses, including SUA and serum creatinine measurements, were performed under fasting conditions within 24 h following IVT treatment, adhering to standardized protocols. This methodological approach aligns with a prior study also examining the association between SUA levels and stroke prognosis (12).

In this study, SUA levels were stratified based on laboratory diagnostic criteria of our hospital, with higher SUA defined as >360 μmol/L and normal SUA as ≤360 μmol/L. While prior studies have employed sex-specific thresholds for hyperuricemia (>420 μmol/L in men and >360 μmol/L in women) (16), accumulating evidence indicates that monosodium urate crystallization can occur at concentrations ≥360 μmol/L, and sustained reduction of serum urate levels below 360 μmol/L promotes crystal dissolution and suppresses disease flares (17). So it has been suggested that an upper threshold value of 360 μmoL/L should be considered for all individuals (18).

Neuroimaging assessments included cranial CT scans at 24 h and MR imaging within 7 days post-IVT to detect both symptomatic and asymptomatic ICH. Neurological deficits were quantitatively evaluated by board-certified clinical neurologists through the standardized application of the NIHSS scores (Range, 0 to 42, with higher scores indicating increased severity of neurological impairment). To ensure objectivity, medical record reviewers were blinded to patient outcomes throughout the data collection process.

### 2.4 Clinical outcomes

The primary outcome was defined as an excellent functional outcome at 90 days post-IVT, characterized by a mRS score of 0 to 1. The mRS is a validated ordinal scale ranging from 0 (indicating no symptoms) to 5 (representing severe disability), with 6 denoting mortality. Secondary outcomes encompassed a favorable functional outcome at 90 days (mRS score 0–2), the cumulative incidence of both symptomatic and asymptomatic ICH within 7 days post-thrombolysis, and 90-day all-cause mortality. The 90-day mRS scores were determined via structured telephone interviews or outpatient evaluations conducted by a board-certified neurologist who remained blinded to the study protocol and treatment allocation.

### 2.5 Statistical analysis

Continuous variables exhibiting a normal distribution were expressed as mean ± standard deviation (SD), whereas those demonstrating non-normal distributions were summarized using median and interquartile range (IQR). Intergroup comparisons between the 90-day mRS classifications (mRS 0–1 group vs. mRS ≥2 group) and SUA levels (higher SUA group vs. normal SUA group) were conducted using the Student’s *t*-test. Categorical variables were expressed as frequency distributions and corresponding percentages, with comparisons performed using either the chi-square (*χ*^2^) test or Fisher’s exact test, as appropriate. Non-parametric comparisons were conducted using the Mann–Whitney U test.

Comparative analyses between higher and normal SUA groups were performed utilizing the Student’s *t*-test for continuous variables and the *χ*^2^ test for categorical variables. Variables demonstrating significant associations (*p* < 0.05) with either the primary outcome (90-day mRS 0–1) or SUA levels were subsequently incorporated into univariate logistic regression models. Significant predictors identified through univariate analysis were then incorporated into a multivariate logistic regression model for further analysis. This adjusted model, incorporating potential confounders such as onset-to-thrombolysis (OTT) time, baseline NIHSS scores and glycated hemoglobin A1c (HbA1c), was employed to evaluate the independent association between SUA levels and the primary outcome, thereby elucidating the relationship between SUA levels and functional outcome at 90 days.

The relationship between SUA levels and secondary outcomes was also examined, with SUA serving as the independent variable. The *χ*^2^ test was employed to assess the relationship between SUA levels and the following variables: 90-day mRS 0–2, post-thrombolysis ICH, and 90-day mortality. Univariate logistic regression analysis was subsequently performed to compare SUA levels with these variables. An adjusted multivariate model was then constructed to evaluate the correlation between SUA levels and secondary outcomes by incorporating significant variables identified from the primary outcomes, including OTT time, baseline NIHSS scores and HbA1c.

Subsequent analyses were performed through hierarchical stratification based on demographic and clinical variables, including age, sex, NIHSS scores, OTT time, atrial fibrillation, post-thrombolysis ICH, hypertension, diabetes mellitus, HbA1c and blood lipid levels. Stratification criteria were defined as follows: age (>70 years vs. ≤70 years), NIHSS score (≥6 vs. <6), OTT (<3 h vs. 3–4.5 h), and HbA1c (≤6.5% vs. >6.5%). The association between SUA levels and the primary outcome was evaluated across subgroups, with adjustments for potential confounding factors, including OTT time, NIHSS scores and HbA1c. All statistical analyses were conducted utilizing R software, version 4.1.2 (R Foundation for Statistical Computing, Vienna, Austria), and statistical significance was determined using a two-tailed *p*-value threshold of <0.05.

## 3 Results

### 3.1 Baseline characteristics differences between higher and normal SUA population

**Figure 1:**
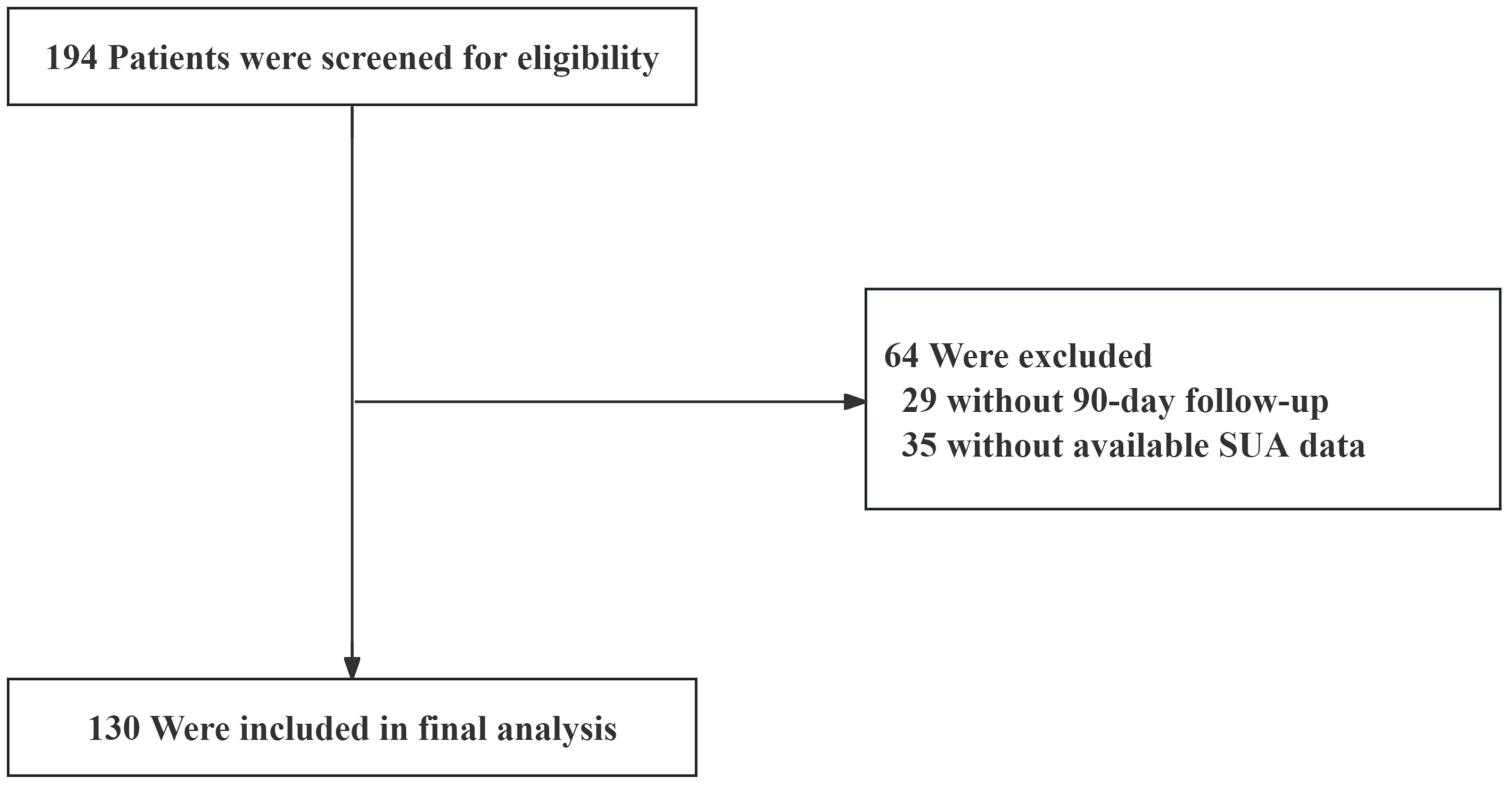
Study flowchart. SUA, serum uric acid.

A cohort of 194 patients admitted to our hospital between November 2021 and December 2024 was initially screened, of which 130 patients who met the inclusion criteria were ultimately included in the final analytical cohort. The remaining 64 patients were excluded based on predetermined exclusion criteria, including 29 patients who were lost to follow-up at the 90-day post-IVT time point and 35 patients with unavailable SUA data (Figure 1).

In the cohort analyzed in the present study, patients with higher SUA levels exhibited significantly younger age (62 ± 10.982 years vs. 67 ± 11.236 years, *p* = 0.010), a higher prevalence of male gender (87.3% vs. 50.7%, *p* < 0.001), and a lower prevalence of diabetes mellitus (27.3% vs. 48%, *p* = 0.019). Additionally, higher SUA levels were significantly associated with decreased high-density lipoprotein cholesterol (HDL-C) concentrations (0.96 ± 0.03 mmol/L vs. 1.08 ± 0.293 mmol/L, *p* = 0.018), elevated urea (6.10 ± 2.294 mmol/L vs. 5.00 ± 1.526 mmol/L, *p* < 0.001) and creatinine levels (82 ± 37.012 μmol/L vs. 69 ± 21.167 μmol/L, *p* < 0.001), diminished estimated glomerular filtration rate (eGFR, 75.41 ± 24.164 mL/min/1.73m^2^ vs. 87.81 ± 23.442 mL/min/1.73m^2^, *p* = 0.004), and lower fasting blood glucose (FBG, 7.80 ± 3.445 mmol/L vs. 9.33 ± 4.190 mmol/L, *p* = 0.029) (Supplementary Table S1).

Following multivariate logistic regression analysis, after adjustment for potential confounding variables, only age [adjusted odds ratio (aOR): 0.973, 95% confidence interval (CI): 0.900–0.987, *p* = 0.012] remained independently and significantly associated with higher SUA levels (Supplementary Table S2).

### 3.2 Association between SUA levels and primary outcome

Among the 130 enrolled patients, 87 (66.92%) achieved an excellent 90-day functional outcome, defined as a mRS score of 0–1, following IVT treatment. Comparative analyses indicated that patients achieving 90-day excellent outcomes exhibited significantly shorter OTT times (142.00 ± 61.33 min vs. 163.00 ± 131.24 min, *p* = 0.001), lower baseline NIHSS scores (4.00 ± 3.84 vs. 7.00 ± 5.24, *p* = 0.024), reduced fasting blood glucose (FBG) levels (8.09 ± 3.45 mmol/L vs. 9.90 ± 4.63 mmol/L, *p* = 0.013), lower glycated hemoglobin A1c (HbA1c) values (6.57 ± 1.38% vs. 7.58 ± 2.19%, *p* = 0.002), and a higher prevalence of hyperuricemia (49.43% vs. 27.91%, *p* = 0.024) (Table 1).

**Table 1:** Demographic and clinical characteristics stratified by 90-day mRS.

Characteristics	All (*N* = 130)	mRS 0–1 (*N* = 87)	mRS 2–6 (*N* = 43)	*p*
Age (years)	65.00 ± 11.38	64.00 ± 11.10	71.00 ± 12.82	0.278
BMI(Kg/m^2^)	24.3 ± 3.57	23.84 ± 3.74	25.25 ± 3.16	0.314
OTT (min)	151.00 ± 113.11	142.00 ± 61.33	163.00 ± 131.24	0.001
DNT (min)	34.50 ± 15.46	34.00 ± 13.89	35.00 ± 17.78	0.346
Baseline NIHSS	4.00 ± 4.12	4.00 ± 3.84	7.00 ± 5.24	0.024
FBG (mmol/L)	8.68 ± 3.95	8.09 ± 3.45	9.90 ± 4.63	0.013
HbA1c (%)	6.89 ± 1.74	6.57 ± 1.38	7.58 ± 2.19	0.002
SUA (mmol/L)	331.00 ± 114.92	355.00 ± 116.37	314.50 ± 110.05	0.141
TC (mmol/L)	4.75 ± 1.32	4.74 ± 1.30	4.85 ± 1.29	0.953
TG (mmol/L)	1.44 ± 0.91	1.46 ± 0.78	1.17 ± 0.97	0.626
HDL-C (mmol/L)	1.02 ± 0.30	1.03 ± 0.31	1.01 ± 0.30	0.538
LDL-C (mmol/L)	3.12 ± 1.09	3.10 ± 1.10	3.03 ± 0.97	0.991
Urea (mmol/L)	5.40 ± 1.99	5.50 ± 2.16	5.00 ± 1.52	0.106
Creatinine (μmol/L)	75.00 ± 31.02	77.00 ± 33.97	72.00 ± 22.94	0.088
eGFR (mL/min/1.73m^2^)	82.57 ± 24.44	80.66 ± 24.53	86.43 ± 24.08	0.206
Urea nitrogen/ creatinine ratio (mg/mg)	16.97 ± 5.60	16.89 ± 5.84	16.98 ± 5.14	0.912
Male	86 (66.15%)	61 (70.11%)	25 (58.14%)	0.237
Hyperuricemia	55 (42.31%)	43 (49.43%)	12 (27.91%)	0.024
Hypertension	84 (64.62%)	52 (59.77%)	32 (74.42%)	0.121
Diabetes	51 (39.23%)	30 (34.48%)	21 (48.84%)	0.130
Atrial fibrillation	9 (6.98%)	5 (5.81%)	4 (9.30%)	0.480
Coronary heart disease	10 (7.69%)	8 (9.20%)	2 (4.65%)	0.495
Post-thrombolysis ICH	10 (7.69%)	4 (4.60%)	6 (13.95%)	0.081

The data are expressed as mean±SD or *n* (%). BMI, body mass index; DNT, door-to-needle time; eGFR, estimated glomerular filtration rate; FBG, fasting blood glucose; HbA1c, glycated hemoglobin A1c; HDL-C, high -density lipoprotein cholesterol; ICH, intracranial hemorrhage, including symptomatic and asymptomatic ICH; LDL-C, low-density lipoprotein cholesterol; mRS, modified Rankin Scale; NIHSS, National Institutes of Health Stroke Scale; OTT, onset-to-thrombolysis time; TC, total cholesterol; TG, triglyceride.

**Figure 2:**
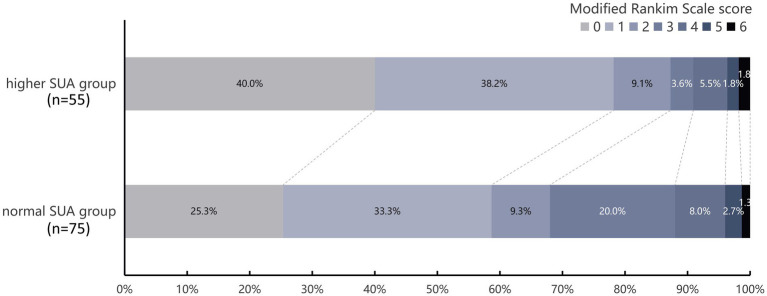
Distribution of modified Rankin scale scores at 90 days post-thrombolysis stratified by serum uric acid levels. Scores range from 0 to 6, with 0 indicating no disability, higher scores indicating greater disability, and 6 indicating death. Higher SUA was defined as >360 μmol/L and normal SUA as ≤360 μmol/L.

The primary outcome analysis revealed that 78.2% of patients in the higher SUA group achieved an excellent 90-day functional outcome (mRS 0–1), which was significantly higher than the 58.7% observed in the normal SUA group (*p* = 0.024) (Table 2; Figure 2). Multivariate logistic regression analysis further substantiated that higher SUA levels were independently associated with an increased likelihood of achieving an excellent 90-day functional outcome (aOR: 2.690, 95% CI: 1.082–6.685, *p* = 0.033) (Table 2).

**Table 2:** Association between different serum uric acid (SUA) levels and outcomes.

Clinical outcomes	Serum uric acid		*p*	OR (95% CI)	*p*	Adjusted OR (95% CI)	*p*
	Normal SUA group	Higher SUA group					
Primary outcome, *n* (%)							
90-day mRS 0–1	44 (58.7%)	43 (78.2%)	0.024	2.525 (1.148, 5.551)	0.021	2.690 (1.082, 6.685)	0.033
Secondary outcomes, *n* (%)							
90-day mRS 0–2	51 (68.0%)	48 (87.3%)	0.013	3.227 (1.274, 8.175)	0.014	4.271(1.438, 12.686)	0.009
Post-thrombolysis ICH	4 (5.3%)	6 (10.9%)	0.321	0.460 (0.123, 1.716)	0.248	0.362(0.076, 1.716)	0.201
90-day mortality	1 (1.3%)	1 (1.8%)	1.000	0.730 (0.045, 11.927)	0.825	—	—

ICH, intracranial hemorrhage, including symptomatic and asymptomatic ICH; mRS, modified Rankin Scale. Hgiher SUA was defined as >360 μmol/L and normal SUA as ≤360 μmol/L. Given the low mortality rates observed in both groups, the calculation of adjusted odds ratios and corresponding *p*-values was precluded following adjustment for potential confounding variables.

Additionally, multivariate logistic regression analysis revealed that prolonged OTT (aOR: 0.993, 95% CI: 0.986–0.999, *p* = 0.044), higher baseline NIHSS scores (aOR: 0.882, 95% CI: 0.796–0.976, *p* = 0.015) and increased HbA1c levels (aOR: 0.762, 95% CI: 0.597–0.972, *p* = 0.029) were independently associated with a reduced probability of achieving an excellent 90-day functional outcome after adjustment for potential confounders (Table 3).

**Table 3:** Multivariate logistic regression analysis of factors that affect the primary outcome.

Characteristics	Reference	Unadjusted model		Adjusted model	
		OR (95% CI)	*p*	OR (95% CI)	*p*
Normal SUA group	Higher SUA group	2.525 (1.148, 5.551)	0.021	2.690 (1.082, 6.685)	0.033
OTT	—	0.991 (0.985, 0.998)	0.007	0.993 (0.986, 0.999)	0.044
Baseline NIHSS	—	0.905 (0.828, 0.989)	0.028	0.882 (0.796, 0.976)	0.015
HbA1c	—	0.702(0.581, 0.898)	0.003	0.762 (0.597, 0.972)	0.029

HbA1c, glycated hemoglobin A1c; NIHSS, National Institutes of Health Stroke Scal; OTT, onset-to-thrombolysis time; Higher SUA was defined as >360 μmol/L and normal SUA as ≤360 μmol/L.

### 3.3 Association between SUA levels and secondary outcome

Regarding the secondary outcome, a significantly higher proportion of AIS patients in the higher SUA group achieved favorable functional outcomes, as defined by a mRS score of 0–2 at 90 days, compared to the normal SUA group (87.3% vs. 68.0%, *p* = 0.013, Table 2; Figure 2). This association was further substantiated by multivariate logistic regression analysis, which demonstrated that higher SUA levels were independently associated with increased likelihood of achieving favorable 90-day functional outcomes (aOR: 4.271, 95% CI: 1.438–12.686, *p* = 0.009). However, no statistically significant associations were observed between SUA levels and either post- thrombolysis ICH or 90-day mortality (Table 2).

### 3.4 Subgroup analyses of the primary outcome

**Figure 3:**
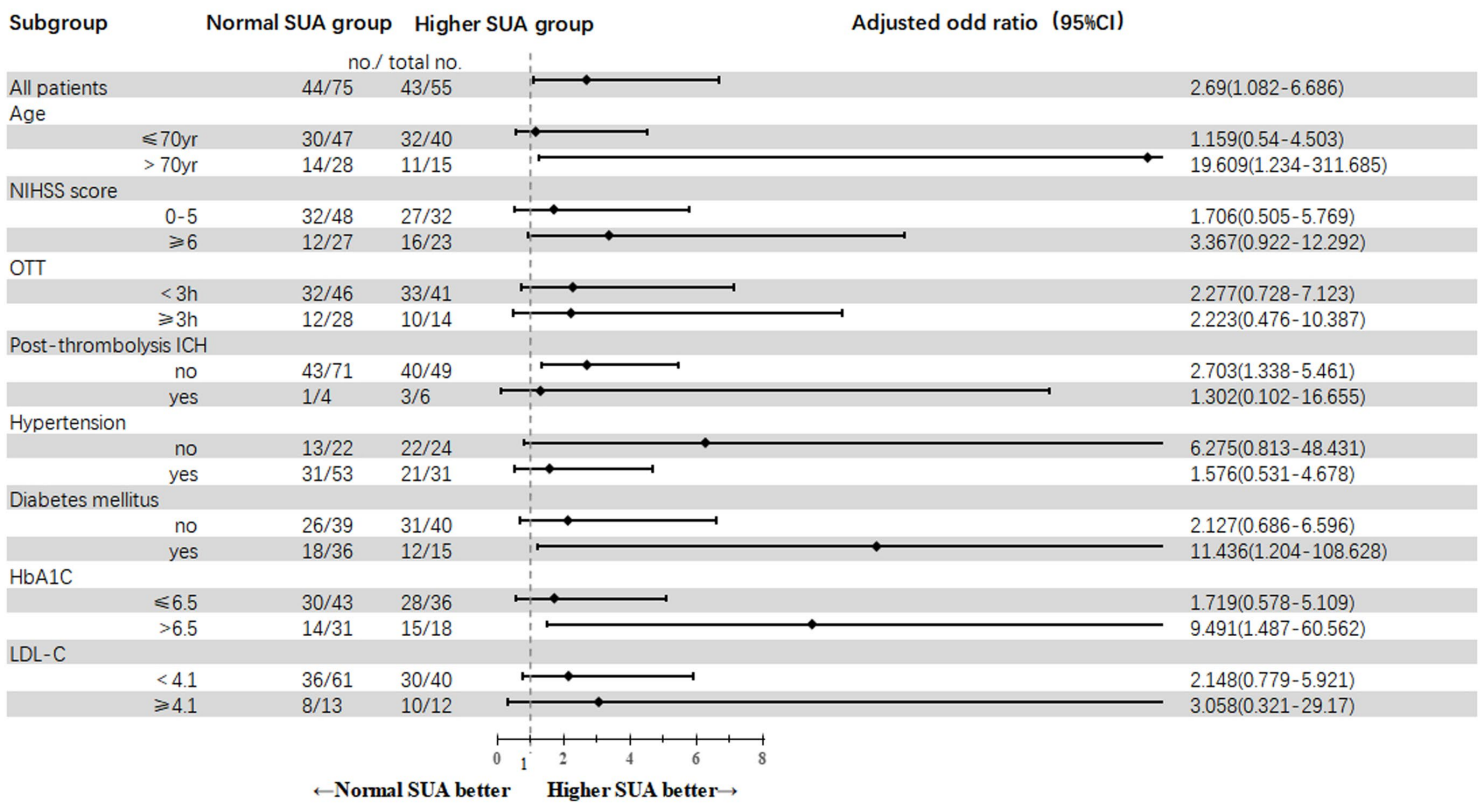
Subgroups analysis of a modified Rankin Scale of 0 to 1 at 90 days (primary outcome). Scores on the National Institutes of Health Stroke Scale (NIHSS) range from 0 to 42, with higher scores indicating greater neurologic deficits. HbA1c, glycated hemoglobin A1c; ICH, intracranial hemorrhage, including symptomatic and asymptomatic ICH; LDL-C, low-density lipoprotein cholesterol; mRS, modified Rankin Scale; OTT, onset-to-thrombolysis; higher SUA was defined as >360 μmol/L and normal SUA as ≤360 mol/L.

Through stratified subgroup analyses based on demographic and clinical characteristics, we observed that patients in the higher SUA cohort demonstrated a significantly increased probability of achieving excellent 90-day functional outcomes when presenting with the following profile: age > 70 years, absence of post- thrombolytic ICH, pre-existing diabetes mellitus and HbA1c levels exceeding 6.5% (Figure 3).

## 4 Discussion

Hyperuricemia is prevalent among ischemic stroke patients in China (7). Analogous to established vascular risk factors such as hypertension and hyperglycemia (15), hyperuricemia has been implicated in the increased incidence of stroke. A meta- analysis of 770,532 adults demonstrated that hyperuricemia is significantly associated with an increased incidence of stroke (19). Paradoxically, both preclinical (20, 21) and clinical studies (13) have indicated that uric acid supplementation exhibits neuroprotective effects in ischemic stroke. These findings suggest that higher SUA levels may exert dual roles in modulating both the incidence and outcomes of ischemic stroke.

A recent prospective cohort study demonstrated that higher SUA levels were positively associated with better clinical outcomes in AIS patients due to large vessel occlusion (LVO) following endovascular treatment (12). Similarly, a clinical study involving AIS patients who underwent IVT and/or endovascular thrombectomy revealed that higher SUA levels at admission were associated with favorable 3-month functional outcomes post-reperfusion therapy, whereas a significant decline in SUA levels was linked to poorer outcomes (22). These findings collectively suggest that higher SUA levels present in the acute phase of ischemic stroke may exert a neuroprotective effect, potentially influencing patient prognosis. However, existing researches have predominantly focused on patients receiving endovascular treatment, leaving the association between SUA levels and clinical outcomes in patients treated exclusively with IVT insufficiently explored.

Our retrospective observational study demonstrated that baseline higher SUA levels were independently associated with a significantly higher likelihood of achieving an excellent functional outcome (mRS 0–1) at 90 days in AIS patients, who underwent alteplase thrombolysis within 4.5 h of stroke onset, after adjusting for potential confounders. Notably, higher SUA levels did not exhibit significant associations with the risk of ICH post-thrombolysis or 90-day mortality, consistent with findings reported in endovascular-treated cohorts (12, 22). Moreover, patients with higher SUA levels demonstrated a significantly greater proportion of favorable 90-day outcomes (mRS 0–2) compared to those with normal SUA levels.

The neuroprotective mechanism of higher SUA in ischemic stroke patients undergoing reperfusion therapy remains incompletely understood. SUA, as the terminal metabolite of purine catabolism, serves as a potent endogenous free radical scavenger (23). Paradoxically, its metabolic generation process concomitantly produces reactive oxygen species (ROS), potentially exacerbating oxidative stress (24). This dualistic nature endows SUA with both pro-inflammatory and antioxidant properties. Preclinical studies have demonstrated that uric acid functions as a robust endogenous antioxidant, effectively neutralizing peroxynitrite toxicity and other reactive species within the neurovascular unit during and following acute cerebral ischemia (25). Conversely, clinical studies in coronary artery disease populations have identified hyperuricemia as a synergistic factor promoting atherogenesis, accelerating plaque progression, and increasing coronary disease burden (26). Our subgroup analysis further revealed that both a history of diabetes mellitus and HbA1c levels >6.5% were significantly associated with the neuroprotective effects of higher SUA levels on clinical outcomes. We hypothesize that persistent hyperuricemia and hyperglycemia may be correlated with an increased prevalence of intracranial atherosclerotic stroke, potentially rendering these patients more responsive to alteplase thrombolysis compared to other stroke etiologies in AIS, as supported by subgroup analyses in a clinical trial (27). However, further investigation is warranted to substantiate these findings.

Notably, our cohort analysis revealed that higher SUA levels were significantly correlated with a lower prevalence of diabetes and reduced fasting blood glucose, which contrasts with recent clinical studies reporting a positive association between hyperuricemia and impaired glucose metabolism (28, 29). This inconsistency may stem from potential selection bias in our single-center study design, given that the median age of the higher SUA group was younger than that of the normal SUA group.

Furthermore, while recent clinical investigations have suggested that higher SUA levels may increase the risk of ICH and subarachnoid hemorrhage (SAH) (30), our findings, consistent with a prior report (12), demonstrated no statistically significant association between higher SUA levels and post-thrombolysis ICH incidence. This discrepancy might also be attributed to the constrained sample size of our study and variations in study design.

Finally, our cohort analysis demonstrated that higher SUA levels were significantly associated with increased serum urea and creatinine concentrations, as well as decreased eGFR. Existing evidence suggests that hyperuricemia may promote renal dysfunction through pathophysiological mechanisms, including chronic inflammation, endothelial dysfunction, and overactivation of the renin-angiotensin-aldosterone system (RAAS) (31). However, subsequent multivariate logistic regression analysis revealed no independent or statistically significant association between these renal function parameters and higher SUA levels after adjusting for potential confounders in our study.

Nevertheless, this study has several notable limitations. Firstly, the study was limited by a relatively small sample size and its single-center design, which could restrict the external validity of the results. Notably, the study excluded patients eligible for endovascular treatment due to LVO, a population previously reported to exhibit an association between SUA levels and clinical outcomes (12). Given that non-LVO etiologies account for an estimated 54%–76% of AIS cases, as evidenced by population-based studies and large clinical registries (32–34), further investigation is warranted to elucidate the relationship between SUA and outcomes in this predominant subgroup. Additionally, randomized controlled trials (RCTs) suggest that non-LVO patients derive greater benefit from IVT than LVO patients. For instance, secondary analyses of the ACT trial demonstrated that only 29.6% of LVO patients achieved an excellent 90-day functional outcome (mRS 0–1) following alteplase administration within 4.5 h of symptom onset (35), whereas the TRACE2 trial reported a significantly higher rate of 60.1% in non-LVO patients (5). Consequently, evaluating the influence of SUA on IVT efficacy in non-LVO patients may hold greater clinical relevance.

Secondly, as a retrospective observational study, we were unable to monitor dynamic changes in SUA levels during the 90-day follow-up period post-discharge, precluding analysis of the impact of SUA fluctuations on functional outcomes. In the Chinese context, socioeconomic factors including patients’ geographical remoteness and limited financial resources frequently result in missed follow-up appointments, which hindered our capacity to obtain longitudinal SUA measurements. Future prospective studies incorporating serial SUA assessments are warranted to elucidate the temporal association between SUA dynamics and clinical outcomes. Additionally, due to limited health literacy among many patients and their families, most participants in our cohort failed to adhere to prescribed medications for managing hypertension, dyslipidemia, hyperglycemia, and hyperuricemia prior to stroke onset, or were unable to provide accurate medication histories. Consequently, this study encountered insufficient data availability for conducting meaningful statistical analyses regarding these pharmacological exposures. Moreover, recent evidence indicates that XO inhibition may confer neuroprotection through purine salvage pathways in ischemic stroke, and serum XO concentration at admission may be an independent risk factor for the onset of acute ischemic stroke, as well as for its progression and prognosis (36). So, further mechanistic studies should be needed to explore whether SUA elevation reflects XO inhibition or other protective pathways.

Thirdly, the limited sample sizes of certain subgroups in the subgroup analysis may increase the risk of type I errors, thereby compromising the reliability of the findings. Besides, the study exclusively focused on patients treated with alteplase within the 4.5-h therapeutic window, leaving the generalizability of these findings to patients receiving alternative thrombolytic agents (e.g., tenecteplase) or treatment beyond 4.5 h uncertain. These limitations underscore the necessity for prospective, multicenter studies with larger cohorts to rigorously validate and further elucidate our observations.

In conclusion, our results reveal a significant positive correlation between higher SUA levels and improved 90-day functional outcomes in non-large vessel occlusion AIS patients undergoing alteplase thrombolysis within the 4.5-h therapeutic window. These findings suggest that SUA may serve as a potential prognostic biomarker in this patient cohort; however, additional evidence is required to establish a causal relationship between SUA levels and clinial outcomes. Therefore, further validation through prospective, multicenter randomized controlled trials with expanded sample sizes is required to substantiate these preliminary observations in the future.

## Data Availability

The raw data supporting the conclusions of this article will be made available by the authors, without undue reservation.
