# Supplementary material for: Higher serum uric acid levels are associated with improved outcomes in acute ischemic stroke patients following intravenous thrombolysis with alteplase—a retrospective cohort study
Source: Front Neurol. 2026 Mar 17;17:1759799. doi: 10.3389/fneur.2026.1759799 (PMC13036852; doi:10.3389/fneur.2026.1759799)
Supplement: Supplementary file 1 [file Data_Sheet_1.docx]

**Supplemental materials:**

Table S1 Baseline demographic and clinical characteristics according to serum uric acid (SUA) level

| Characteristics | Serum uric acid | | P |
| --- | --- | --- | --- |
|  | Normal SUA group | Higher SUA group |  |
| Age (years) | 67±11.236 | 62±10.982 | 0.010 |
| Male | 38 (50.7%) | 48 (87.3%) | <0.001 |
| OTT (min) | 160±136.889 | 141±66.594 | 0.170 |
| DNT (min) | 34±15.384 | 35±15.698 | 0.946 |
| Baseline NIHSS | 4±3.502 | 5±4.790 | 0.133 |
| FBG(mmol/L) | 9.33±4.190 | 7.80±3.445 | 0.029 |
| HbA1c(%) | 7.14±2.012 | 6.55±1.207 | 0.055 |
| TC (mmol/L) | 4.71±1.211 | 4.90±1.473 | 0.891 |
| TG (mmol/L) | 1.36±0.884 | 1.58±0.940 | 0.243 |
| HDL-C (mmol/L) | 1.08±0.293 | 0.96±0.030 | 0.018 |
| LDL-C (mmol/L) | 3.04±1.007 | 3.33±1.205 | 0.958 |
| Urea (mmol/L) | 5.00±1.526 | 6.10±2.294 | <0.001 |
| Creatinine (μmol/L) | 69±21.167 | 82±37.012 | <0.001 |
| eGFR(mL/min/1.73m^2^) | 87.81±23.442 | 75.41±24.164 | 0.004 |
| Urea nitrogen/ creatinine ratio (mg/mg) | 18.20±5.799 | 16.04±5.304 | 0.280 |
| Hypertension | 53 (70.7%) | 31 (56.4%) | 0.099 |
| Diabetes | 36 (48%) | 15 (27.3%) | 0.019 |
| Atrial fibrillation | 6 (8%) | 3 (5.6%) | 0.734 |
| Coronary heart disease | 3 (4%) | 7 (12.7%) | 0.095 |
| Post-thrombolysis ICH | 4 (5.3%) | 6 (10.9%) | 0.321 |

The data are expressed as mean±SD or n (%). Abbreviation: BMI, body mass index; DNT, door-to-needle time; eGFR, estimated glomerular filtration rate; FBG, fasting blood glucose; HbA1c, glycated hemoglobin A1c; HDL-C, high -density lipoprotein cholesterol; ICH, intracranial hemorrhage, including symptomatic and asymptomatic ICH; LDL-C, low-density lipoprotein cholesterol; mRS, modified Rankin Scale; NIHSS, National Institutes of Health Stroke Scale; OTT, onset-to-thrombolysis time; TC, total cholesterol; TG, triglyceride. Hgiher SUA was defined as >360 μmol/L and normal SUA as ≤360 μmol/L.

Table S2 Multivariate analysis of the factors that affect SUA level

| Characteristics | Serum uric acid (μmol/L) | | |
| --- | --- | --- | --- |
|  | Elevated SUA group | Normal SUA group | |
|  |  | Adjusted-OR（95%CI） | P |
| Age | Reference | 0.973(0.900, 0.987) | 0.012 |
| FBG | Reference | 0.954(0.804, 1.132) | 0.591 |
| HDL-C | Reference | 0.192 (0.024, 1.564) | 0.123 |
| Urea | Reference | 1.399 (0.988, 1.983) | 0.059 |
| Creatinine | Reference | 1.011 (0.959, 1.066) | 0.689 |
| eGFR | Reference | 0.980 (0.930, 1.033) | 0.455 |
| Male | Reference | 2.735 (0.624, 11.991) | 0.182 |
| Diabetes mellitus | Reference | 3.282 (0.839, 12.831) | 0.088 |

Hgiher SUA was defined as >360 μmol/L and normal SUA as ≤360 μmol/L. Abbreviation: eGFR, estimated glomerular filtration rate; FBG, fasting blood glucose; HDL-C, high -density lipoprotein cholesterol.
